# Supplementary material for: Beneficial Effects of In Vitro Reconstructed Human Gut Microbiota by Ginseng Extract Fermentation on Intestinal Cell Lines
Source: Microorganisms. 2025 Jan 17;13(1):192. doi: 10.3390/microorganisms13010192 (PMC11768043; doi:10.3390/microorganisms13010192)
Supplement: Supplementary file 1 [file microorganisms-13-00192-s001.zip › microorganisms-3405034-supplementary.pdf]

Table S1. Bacterial primer sets utilized in the study.

Species	Primer code	Sequence	Target	Reference
L. plantarum	Lpl2F<br>Lpl2R	CATTGGAACCGAACCAGTTG<br>CGGTGTTCTCGGTTTCATTATG	16S/23S IS	Mezzasalma et al., 2016 [26]<br>De Giani et al., 2022 [27]
L. acidophilus	Lacid2F<br>Lacid 2R	GGGCAAATCACGAACGAGTA<br>CTTTGTTTTCGTTCGCTTCA	Pre 16S	Mezzasalma et al., 2016 [26];<br>De Giani et al., 2022 [27]
B. animalis subsp. *lactis*	AnimF<br>AnimR	GCACGGTTTTGTGGCTGG<br>GACCTGGGGGACACACTG	Pre 16S	Mezzasalma et al., 2016 [26];<br>De Giani et al., 2022 [27]
B. cellulosilyticus	BAC_16S_F<br>BAC_16S_R	GGTAGTCCACACAGTAAACGATGAA<br>CCCGTCAATTCCTTTGAGTTTC	16S	Dejean et al., 2020 [28]
F. plautii	Fplautii170F<br>Fplautii423R	GGTCGCATGGCTCTGACT<br>TCATTTGTTTCGTCCCCGAC	16S	Alauzet et al., 2019 [29]
C. symbiosum	CS_F<br>CS_R	GTGAGATGATGTGCCAGGC<br>TACCGGTTGCTTCGTCGATT	hgdA	Xie et al., 2017 [30]
E. coli	EC_F<br>EC_R	CATGCCGCGTGTATGAAGAA<br>CGGGTAACGTCAATGAGCAAA	16S	Huijsdens et al., 2002 [31]
